# The Principles and Practice of Surgery

**Published:** 1863

**Authors:** 


### Book Notices.

The Principles and Practice of Surgery; Embracing Minor and Operative Surgery, with a Bibliographical Index of American Surgical Writers, from the Year 1783 to 1860. Arranged for the use of Students, and Illustrated by 400 Woodcuts and nearly 1000 Engravings on Steel. By Henry H. Smith, M.D., Prof. of Surgery in the University of Pennsylvania, &c. Philadelphia: J. B. Lippincott & Co. 1863.

This work is in two volumes of closely condensed matter, each containing about eight hundred pages. The treatise is by no means entirely new, being a sort of a condensation of three of the author's previous works, viz.: his "*Minor Surgery,*" "*Operative Surgery,*" and "*Practice of Surgery.*" These have been revised and condensed, and several hundred pages of new matter added, to give the whole a complete form. The illustrations are very copious, especially upon the subject of the instruments and operations of surgery.

Prof. Smith and his works are so well known to the country, that it is unnecessary to review this compilation in detail; suffice it to say, that it contains all the excellencies of the three works embodied in it, together with much valuable matter superadded,—the whole constituting a very complete and full text-book for students, and an excellent work of reference for young practitioners.

Considered as an author, Prof. Smith has the merit of being very full and minute in his attention to practical details, and, perhaps, carries it even to a fault, by absorbing the readers' attention a little too much upon minor points, and causing him, to a certain extent, to loose sight of the broader and deeper general principles, which give scope to the thought and fertility to the resources of the young surgeon. Considered in a purely literary point, his style of writing is slightly rough, from the selection of an excess of words containing strong consonants, and his sentences are a little overloaded with polysyllables of Latin derivation. He is, however, perfectly clear in his expressions, and orderly in his arrangement; and his rare faculty of condensation enables him, in spite of a little excess of long words, to express more ideas within a small space than any prominent surgical author on this side of the Atlantic.

The work is sold for twelve dollars; and will be a valuable addition to the library of all who wish to bring down their collection of surgical works to the latest date.

